# A late, solitary brain metastasis of epithelial ovarian carcinoma

**DOI:** 10.1186/1471-2407-14-543

**Published:** 2014-07-28

**Authors:** Raffaele Longo, Christian Platini, Nada Eid, Clémence Elias-Matta, Thaar Buda, Denis ’Nguyen, Philippe Quétin

**Affiliations:** Division of Medical Oncology, CHR Metz-Thionville, 1 Allée du Château, 57085 Ars-Laquenexy, France; Division of Radiology, CHR Metz-Thionville, 1 Allée du Château, 57085 Ars-Laquenexy, France; Division of Radiotherapy, CHR Metz-Thionville, 1 Allée du Château, 57085 Ars-Laquenexy, France

**Keywords:** Ovarian cancer, Brain metastases

## Abstract

**Background:**

Brain metastasis from epithelial ovarian cancer (EOC) is very rare with a reported incidence of less than 2%. It is usually associated with a poor prognosis that is related to several factors, the most important including: single *vs* multiple lesions, performance status, platinum-sensitive disease, tumor grade, extracranial disease, and multimodal approach treatment. At the time of diagnosis, an extracranial disease is found in over half of patients. The most common histology is the serous type. The median time from primary diagnosis to development of cerebral lesions is directly correlated to initial tumor grade and stage. Several therapeutic approaches can be proposed, including best supportive care +/- corticosteroids, surgery, radiotherapy and chemotherapy. A multimodal therapy approach may achieve an improved outcome and should therefore be utilized whenever applicable.

**Case presentation:**

We present the case of a patient with a solitary brain metastasis which appeared 11 years after a locally advanced and aggressive EOC (FIGO stage III C) and which totally regressed after surgery and adjuvant chemotherapy. Clinically, she showed progressive headaches, decreased visual acuity, balance and memory disorders associated with a confusional state. Brain CT scan and MRI documented a solitary, necrotic lesion in the left central parietal region with an important cerebral surrounding edema and initial cranial herniation. No other extracranial metastases were observed at the PET scan. Laboratory tests were in the normal range and CA 125 was moderatly increased at 81 UI/ml. The patient underwent surgical removal of tumor lesion, post-surgical whole-brain radiotherapy (WBRT) and systemic chemotherapy with carboplatin alone for six cycles. At a follow-up of 13 months, she is alive, in good clinical condition and tumor progression free.

**Conclusion:**

The peculiarity of this case relies on the isolated brain relapse of a BRCA-1/BRCA-2 non-mutated EOC, which is uncommon and rare, and to the very long time, of 11 years, from diagnosis of primary cancer and development of brain metastasis. A multimodal, aggressive approach of this isolated brain metastasis led to a complete and prolonged tumor control.

## Background

Approximately 20–40% of all cancer patients develop brain metastases with breast cancer, lung cancer and melanoma representing the most common primary tumors [1]. The reported incidence of brain metastases from epithelial ovarian cancer (EOC) is less than 2%. Brain involvement is usually associated with a poor prognosis [1–3]. Several factors seem to have an impact on overall survival (OS), including single lesion, performance status, platinum-sensitive disease, tumor grade, extracranial disease, and, probably, multimodal approach but all these factors have not been evaluated and validated in prospective studies [4, 5]. The standard treatment consists of whole-brain radiotherapy (WBRT), which improves the quality of life by a reduction of neurological symptoms and prolongs OS to 3–6 months [6]. Patients with a good performance status, a controlled extracranial disease and a single brain lesion are generally considered for more aggressive therapeutic strategies, including surgical resection, stereotactic radiosurgery, postoperative radiation, and chemotherapy [7].

We report an interesting case of a 63-year-old woman presenting an isolated EOC brain metastasis, 11 years after the primary tumor treatment.

## Case presentation

**Figure 1:**
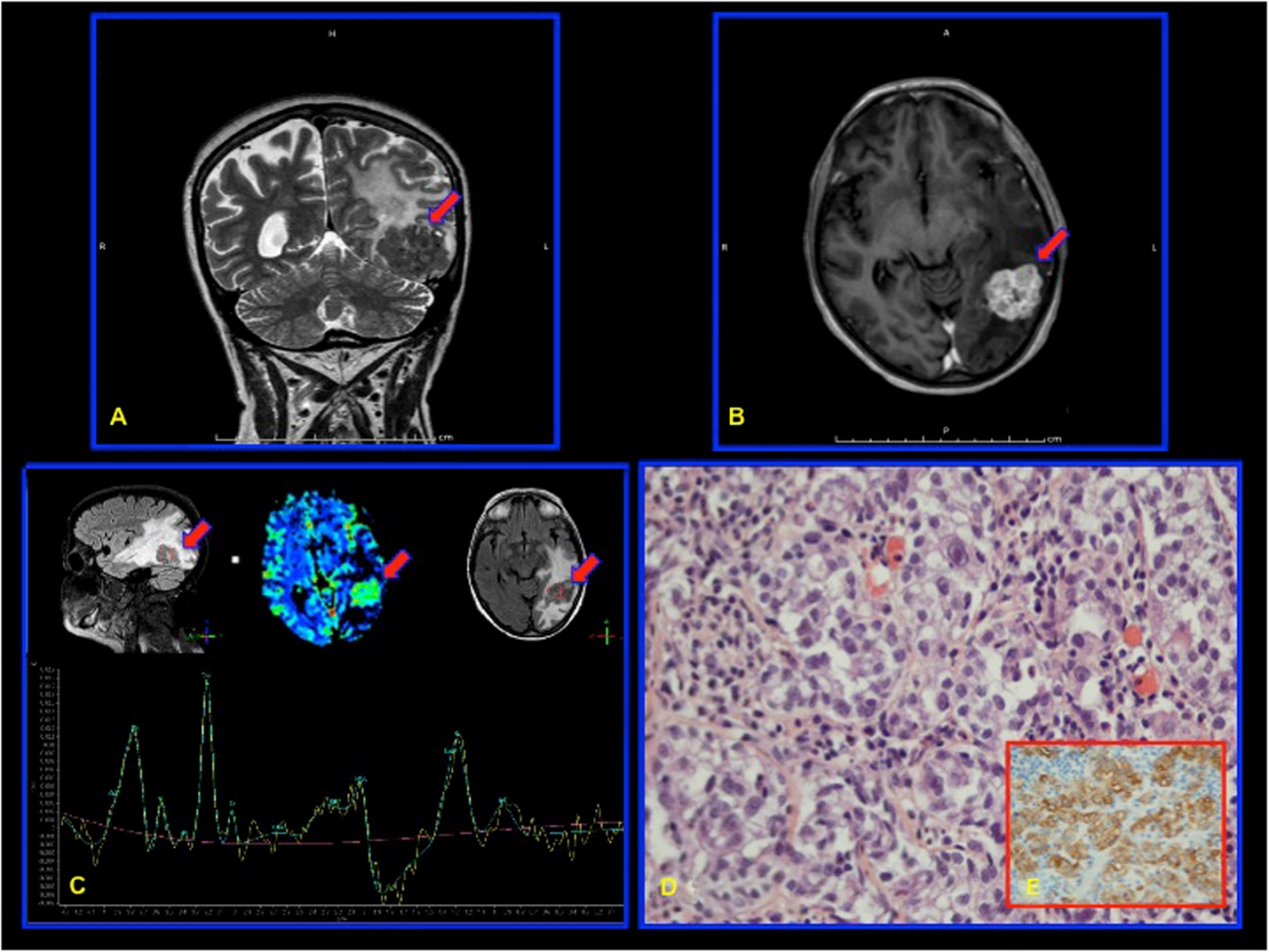
**A solitary brain metastasis of epithelial ovarian carcinoma (EOC). A–B**: A solitary, necrotic lesion in the left central parietal region with an important cerebral surrounding edema and initial cranial herniation (brain MRI; red arrow). **C**: Absence of neurological cells in the tumor lesion (SPECT MRI; red arrow). **D**: A metastatic poorly differentiated carcinoma (Histology). **E**: Cytokeratin 7 and WT1 positivity of tumor cells (Immunohistochemistry).

In Jun 2001, a 51-year-old woman was submitted to radical surgery followed by 6 cycles of systemic chemotherapy with a carboplatin/taxol regimen for a FIGO stage IIIC mixed, undifferentiated and clear-cells ovarian cancer. The follow-up was uneventful until July 2012 when an increase of the tumor marker CA 125 was observed at 46 UI/ml. A chest-abdomen and pelvic CT scan was also performed and it was negative. In December 2012, the patient was hospitalized because of progressive headaches, decreased visual acuity, balance and memory disorders associated with a confusional state. Brain CT scan and MRI documented a solitary, necrotic lesion in the left central parietal region with an important cerebral surrounding edema and initial cranial herniation (Figure 1A-B, red arrow). A PET scan was negative. Laboratory tests were in the normal range and CA 125 was at 81 UI/ml. The SPECT MRI revealed the absence of neurological cells in the lesion leading to the hypothesis of a late, solitary ovarian metastasis (Figure 1C, red arrow).

A corticosteroid therapy was started with a progressive improvement of neurologic symptoms. In January 2013 she underwent craniotomy with radical tumor resection. Histology showed a metastatic poorly differentiated carcinoma with an immunohistochemical profile (cytokeratin 7/WT1 positive) compatible with metastasis from ovarian primary (Figure 1D-E). A post-surgery WBRT has been performed followed by a systemic monochemotherapy by carboplatin for six cycles.

A mutational analysis was also performed and it did not find any mutation of the BRCA1/BRCA2 genes.

At a follow-up of 13 months, the patient is alive, asymthomatic, in good clinical conditions and tumor progression free. The CA 125 is in the normal range (29 UI/ml).

## Conclusions

Brain metastases from EOC are very uncommon with a reported incidence in the literature of 1–2.5% [1–3]. This incidence is probably underestimated because of the fact that brain imaging is not generally included in routine follow-up. However, from its first description in 1978 by Mayer et al. [8], a rise in incidence has been observed probably reflecting: i) an improvement of imaging techniques, leading to an earlier and more sensitive diagnosis of brain lesions; ii) a prolonged patients’ survival by a better therapeutic strategy; iii) biological mechanisms involving the blood–brain barrier (BBB) that can either lead to an early brain implantation after its potential chemotherapy-induced imparement or avoid a drug penetration into the brain acting as a safe haven.

Radiologically, brain involvement is often multiple and presents a mixed, solid-cystic form [9]. The frontal lobe is the most frequent localisation [9]. At time of diagnosis, an extracranial disease is found in over half of the patients [1–3].

About 80% of the patients present a FIGO stage III or IV at the first diagnosis of EOC [2]. The most common histology is the serous type, which is reported in over half of the cases, followed by mixed epithelial, endometrioid, adenocarcinoma, mucinous, undifferentiated and clear cell histology, all in decreasing frequency [2].

The reported median time from primary diagnosis to development of cerebral lesions is variable and directly correlated to initial tumor grade and stage [10, 11].

Only two independent prognostic factors have been shown to have a relevant clinical impact: i) the presence of extracranial disease at the time of brain relapse and ii) the performance status [1]. Conflicting results have been reported with other potential factors, including "single" *vs* "multiple" localisations, the interval time from primary diagnosis and metastatic manifestation, tumor stage, grade, histotype, age at diagnosis of brain relapse, site of lesion, and treatment strategy [4, 12]. However, a recent German, multicenter, retrospective review documented, in a multivariate analysis, five negative prognostic factors, including performance status, platinum-sentive disease, tumor grading, FIGO staging, and presence of multiple *vs* single lesions. Other factors, such as histology, age at primary diagnosis, ascites, residual tumor at primary surgery, extracranial disease at time of brain relapse, presentation with or without headache did not directly impact on OS. A trend for significatly longer survival for the multimodal therapy was also observed [5].

Germlime mutations of BRCA-1 and -2 genes have been reported in 10% of EOC and they seem to correlate with a more aggressive behaviour and a metastatic disease [13]. Recently, several reports showed a strict correlation between BRCA-1 mutations and incidence of brain metastases in EOC [14, 15].

Several therapeutic approaches can be proposed, including best supportive care +/- corticosteroids, surgery, radiotherapy and chemotherapy. When applicable, conventional neurosurgery promises the best results. In case of contraindications to surgery, inaccessibility of lesions and the presence of multiple metastases, the most commonly used individual therapy strategy is WBRT that improves neurological symptoms and prolongs median survival up to 3–6 months [1, 6].

The role of systemic chemotherapy is controversial with a reported median OS up to 16 months even when combined with surgery or radiation [2, 7]. These data probably reflect the impossibility to reach a high cerebrospinal fluid drug concentration with the actual cytotoxic agents most active in EOC which are unable to cross the blood–brain barrier.

Several studies have documented an increased therapeutic potential by a multimodal, more aggressive approach.

Cohen et al. reported a median OS of 5.6 months for surgery or WBRT, when these are employed individually, and of 23.1 months after a combination of both strategies [6]. The addition of chemotherapy to surgery and radiotherapy seems to improve OS of 3–6 months but these data derived from retrospective, small-cohorts studies and have not been prospectively validated [1, 16]. Recently, stereotactic radiotherapy showed promising results with a remarkable median survival of 29 months as compared to 6 months with WBRT [17] but it is mostly applied in selected patients with no more than 3 cerebral lesions. It is now prospectively evaluating into a multimodal approach and also in patients with multiple lesions.

A multimodal therapy approach may achieve an improved outcome for the patients and should therefore be used whenever applicable. The evidence available to date is pointing towards a combination of surgery and radiotherapy as the treatment modality with the best benefit to drawback ratio [1]. An additional chemotherapy can potentially improve clinical outcome in some patients, but this benefit has to be measured against possible side-effects. Further research is needed to clarify the value of chemotherapeutic agents having a potential of reaching high concentrations in the cerebrospinal fluid.

In our case, the patient presented a solitary brain metastasis 11 years after a locally advanced and aggressive EOC which totally regressed after surgery and post-surgical chemotherapy. Brain involvement was associated with a moderatly increase of tumor marker CA125. No other extracranial metastases were observed. The patient underwent a multimodal and aggressive treatment, including surgical resection of the tumor lesion, post-surgical WBRT and systemic chemotherapy with carboplatin alone for six cycles that led to a complete and prolonged tumor control.

The peculiarity of this case relies on the isolated brain relapse of a BRCA-1 and BRCA-2 non-mutated EOC, which is uncommon and rare, and to the very long time, of 11 years, from diagnosis of primary cancer and development of brain metastasis. Finally, a multimodal and aggressive approach achiewed a prolonged tumor-free survival.

## Consent

Written informed consent was obtained from the patient for publication of this Case report and any accompanying images. A copy of the written consent is available for review by the Editor of this journal.
